# A retrospective study of MYC rearranged diffuse large B-cell lymphoma in the context of the new WHO and ICC classifications

**DOI:** 10.1038/s41408-023-00827-5

**Published:** 2023-04-18

**Authors:** Dima El-Sharkawi, Amit Sud, Catherine Prodger, Jahanzaib Khwaja, Rohan Shotton, Brian Hanley, Victoria Peacock, Ying Ying Peng, Anita Arasaretnam, Sarkhara Sharma, Frances Aldridge, Bhupinder Sharma, Andrew Wotherspoon, Betty Cheung, Corinne De Lord, Rosalynd Johnston, Shireen Kassam, Ruth Pettengel, Kim Linton, Paul Greaves, Lucy Cook, Kikkeri N. Naresh, Kate Cwynarski, Toby A. Eyre, Ian Chau, David Cunningham, Sunil Iyengar

**Affiliations:** 1 The Royal Marsden NHS Foundation Trust and the Institute of Cancer Research, London, UK; 2 Division of Genetics and Epidemiology, The Institute of Cancer Research, London, UK; 3 Churchill Hospital, Oxford University Hospitals NHS Foundation Trust, Oxford, UK; 4 University College Hospitals NHS Foundation Trust, London, UK; 5 The Christie Hospital NHS Foundation Trust, Manchester, UK; 6 Imperial College Healthcare NHS Trust, London, UK; 7 King’s College Hospital NHS Foundation Trust, London, UK; 8 St George’s University Hospitals NHS Foundation Trust, London, UK; 9 Brighton and Sussex University Hospitals NHS Trust, Brighton, UK; 10 Croydon Health Services NHS Trust, Croydon, UK; 11 Epsom and St Helier University Hospitals NHS Trust, London, UK; 12 Barking, Havering and Redbridge University Hospitals NHS Trust, Essex, UK

**Keywords:** B-cell lymphoma, B-cell lymphoma, Genetic translocation, Genetic testing, Disease-free survival

**Dear Editor**,

Diffuse large B-cell lymphoma (DLBCL) is an aggressive but biologically heterogenous Non-Hodgkin Lymphoma (NHL). Clinical prognostic scores such as the International Prognostic Index (IPI) are established predictors of outcome. There is however significant interest in using genomics to advance risk prediction as well as inform precision medicine strategies [1–7]. While genomic classification is evolving, chromosomal rearrangements involving the *MYC, BCL2* and *BCL6* are now widely tested for at diagnosis using fluorescence in situ hybridisation (FISH). Patients with *MYC* rearrangements (MYCR) have inferior outcomes and those with MYCR and concomitant translocations of *BCL2* or *BCL6* or both have particularly poor outcomes. The 2016 revision of the World Health Organisation (WHO) classification of haematological neoplasms subsequently categorised these lymphomas as high grade B-cell lymphomas (HGBL) with *MYC, BCL2* and /or *BCL6* rearrangements [8]. However, the recent 2022 iteration of the WHO classification excludes *MYC-BCL6* rearranged cases from this category as these lymphomas are genetically heterogenous and distinct when compared to the *MYC-BCL2* +*/−* *BCL6* rearranged cases [9, 10]. In comparison, the simultaneously released International Consensus Classification (ICC), while also recognising the distinct biology in *MYC-BCL6* rearranged double hit (DH) cases, retains these cases as a sub-category on the basis that some studies have recorded poor outcomes in these patients [11].

In view of the new WHO and ICC classifications, we undertook a UK multicentre retrospective data collection to study the management and outcomes of patients with MYCR DLBCL. *MYC-BCL6* rearranged cases were separated out from *MYC-BCL2* “double hit” or “triple-hit” lymphomas (DH/TH) cases as defined by the new 2022 WHO and ICC recommendations. This retrospective multicentre service evaluation was approved by the committee for clinical research at the Royal Marsden Hospital (SE759) and by the Research and Development departments of individual participating centres. Data were analysed on 220 MYCR DLBCL cases, including DH/TH cases. Cases were identified by cytogenetics departments of hospitals and anonymised clinical data were collated via secure email. Differences between baseline characteristics were tested for using the unpaired t-test, chi-squared test or the Mann–Whitney *U* test. Time to next treatment (TTNT) was defined as the interval from diagnosis to the start of second-line therapy or death, whichever occurred first [12]. Patients treated with either dose-adjusted R-EPOCH (DA-R-EPOCH) or R-CODOX-M/R-IVAC were included in the ‘intensified’ chemotherapy group. Patients receiving ≤2 cycles of initial R-CHOP treatment prior to intensification, once FISH results were available, were included in this group. Overall survival (OS) was defined as the time between diagnosis and death from any cause. For TTNT and OS, patients were censored at the latest date known to be alive or at the end of the study period. 5-year TTNT and 5-year OS were estimated using the Kaplan-Meier method [13]. TTNT and OS curves were compared using the log-rank test [14]. Median follow-up time was estimated using a reverse Kaplan-Meier estimator [15]. Cox proportional hazards regression was used to assess the association of baseline characteristics and outcome [16]. Univariate and multivariate models were used to estimate hazard ratios (HRs) and their 95% confidence intervals (CI) with proportionality of hazards confirmed by Schoenfeld residuals [17]. *P-*values <0.05 were considered statistically significant. All analyses were performed using R statistical software using survival and survminer packages [18].

Patient data were returned on 220 patients from 16 UK centres. Comprehensive characteristics of patients are reported in Table 1. Among patients with DH/TH, a significantly higher proportion (*P* = 0.01) presented with stage 3 or 4 disease (92/105) when compared to MYCR and MYC-BCL6 cases (48/66) (Supplementary Table 1). Intensified therapy was given to 32% of DH, TH, *MYC*-*BCL6* and MYCR cases treated with curative intent. Patients who received intensive therapy were younger (*P* = 0.009) and had a more advanced stage at diagnosis (*P* = 0.01) (Supplementary Table 1). The median follow‐up by reverse censoring was 30 months. One hundred and four patients had died, and 86 had received a second treatment regimen during follow-up. The median OS for the entire cohort was 30 months (Fig. 1A). Restricting the analysis to patients receiving chemotherapy, median OS was 42 months with a median TTNT of 12 months (Fig. 1B, C).

**Table 1:** Patient characteristics.

Characteristic		All patients (*n* = 220)
Age	Median (years)	66
	Range (years)	17–97
Age groups (years)	10–29	3
	30–49	23
	50–69	112
	70–89	77
	>90	5
Sex	Male	131
	Female	89
Performance status	0–1	113
	≥2	67
	Missing	40
Stage	1–2	39
	3–4	152
	Missing	29
Lactate dehydrogenase	<Upper limit of normal	37
	>Upper limit of normal	129
	Missing	54
CNS involvement at diagnosis	Present	6
CNS involvement at relapse	Present	8
International Prognostic Index	0–2	54
	≥3	102
	Missing	64
Double hit	BCL2	84
	BCL6	21
Triple hit		31
*MYC* rearranged	*BCL2*/*BCL6* not tested	24
	MYC-R only confirmed	60
Front-line therapy	RCHOP or RB-CHOP	113
	RGCVP	8
	R-miniCHOP	10
	RCVP	8
	Other	9
	DA-R-EPOCH	19
	R-CODOX-M/IVAC	28
	Palliative	25

Age was considered in 5 categories (10–30 years, 30–50 years, 50–70 years, 70–90 years, 90–110 years); Eastern Cooperative Oncology Group (ECOG) performance status (PS) in 2 categories (0–1, 2–5); stage in 2 categories (1–2, 3–4); lactate dehydrogenase (LDH) in 2 categories (normal, >upper limit of normal (ULN)); and IPI in two categories (low, 0–2; high, 3–5).

**Fig. 1:**
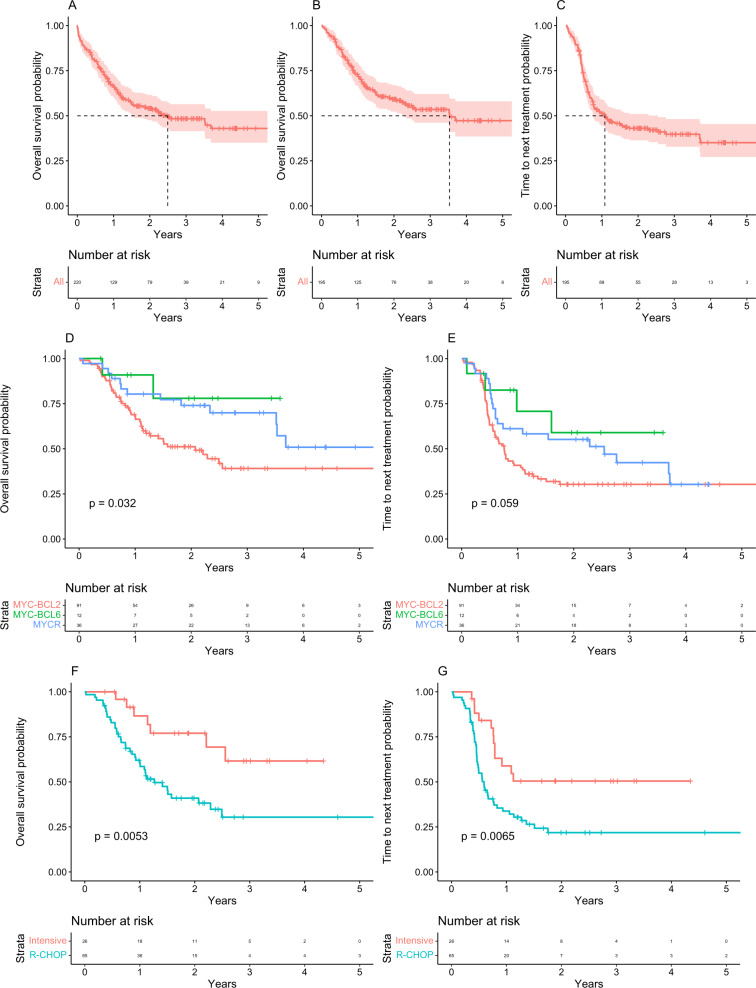
Kaplan–Meier estimates for overall survival and time to next treatment for Myc rearranged DLBCL patients in this study. **A** Overall survival in all patients, including *MYC*-*BCL2* double hit and triple hit, *MYC* rearranged and *MYC*-*BCL6* diffuse large B-cell lymphoma; **B** overall survival for patients with *MYC*-*BCL2* double hit and triple hit, *MYC* rearranged and *MYC*-*BCL6* diffuse large B-cell lymphoma treated with all types of chemoimmunotherapy; **C** time to next treatment for patients with *MYC*-*BCL2* double hit and triple hit, *MYC* rearranged and *MYC*-*BCL6* diffuse large B-cell lymphoma treated with all types of chemoimmunotherapy; **D** overall survival for patients treated with RCHOP or intensive therapy stratified by *MYC*-*BCL2* double hit and triple hit, *MYC* rearranged and *MYC*-*BCL6* translocation status; **E** time to next treatment for patients treated with R-CHOP or intensive therapy stratified by *MYC*-*BCL2* double hit and triple hit, *MYC* rearranged and *MYC*-*BCL6* translocation status; **F** overall survival in all patients with *MYC*-*BCL2* double hit and triple hit diffuse large B-cell lymphoma stratified by treatment with RCHOP or intensive therapy; **G** time to next treatment in all patients with *MYC*-*BCL2* double hit and triple hit diffuse large B-cell lymphoma stratified by treatment with RCHOP or intensive therapy.

When comparing MYCR vs *MYC*-*BCL6* vs *MYC*-*BCL2* DH/TH DLBCL cases that were initiated on chemoimmunotherapy, the probability of survival was significantly lower in *MYC*-*BCL2* DH/TH DLBCL with most deaths occurring within the first 2 years of diagnosis (Fig. 1D, E). Significant differences were seen in TTNT and OS when comparing patients with *MYC*-*BCL2* DH/TH DLBCL treated with intensified chemotherapy vs R-CHOP—respective median OS not reached (NR) (95% CI: 30 months-NR) vs 15 months (95% CI: 12–30 months), HR 3.0, *P* = 5.3 × 10^−3^; and median TTNT NR (95% CI: 9 months-NR) vs 7 months (95% CI: 6–10 months), HR 2.35, *P* = 6.5 × 10^−3^ (Fig. 1F, G). Patients with *MYC*-*BCL2* DH/TH treated with intensified chemotherapy had a lower median age (56 years vs 65 years, *P* = 9 × 10^−3^) and better performance status (*P* = 0.05) (Supplementary Table 1). Treatment intensification was associated with a lower TTNT in *MYC*-*BCL6* DLBCL but interpretation is limited by the small number of cases and events (Supplementary Fig. 1). Outcomes in MYCR DLBCL were not affected by treatment intensification (Supplementary Fig. 2). TTNT and OS were explored in a univariate non‐stratified Cox regression model by baseline characteristics. Older age, advanced stage (≥3), ECOG PS ≥ 3, LDH > ULN and a high IPI group (≥3) were associated with inferior OS and ECOG PS ≥ 3, LDH > ULN and a high IPI group (≥3) were associated with inferior TTNT (Supplementary Table 2). In a multivariate Cox-regression model incorporating age, age and PS or IPI score, intensive treatment remained significantly associated with OS and TTNT in patients with *MYC*-*BCL2* DH/TH (Supplementary Tables 3 and 4). OS and TTNT in patients receiving platinum-based chemotherapy for relapsed or refractory disease were poor with no significant difference observed between DH/TH, *MYC*-*BCL6* and MYCR DLBCL (*P* = 0.99, median OS 7.2 months, 4 months and 15 months respectively) (Supplementary Fig. 3).

The aim of this large UK retrospective study was to analyse the management and outcomes of patients with MYCR DLBCL including *MYC*-*BCL2* DH/TH DLBCL with reference to the new 2022 WHO and ICC definitions. The median age at presentation in this study was similar to previously published data but lower than expected for DLBCL perhaps reflecting a bias in FISH testing for younger patients [19]. The majority of patients with *MYC*-*BCL2* DH/TH DLBCL had advanced-stage disease at diagnosis, consistent with previously published data and different to those with MYCR DLBCL [19–22].

Since the publication of the UK NICE guidance in 2005, FISH testing in DLBCL is widely performed. There are however variations in testing criteria with some centres performing FISH in all patients with DLBCL, while others are more selective, restricting testing to DLBCL patients that have >40% MYC expression on their biopsies. Furthermore, FISH testing for *MYC* translocation partner genes is not widely available in the UK. Such data was therefore absent in the majority of cases in this study.

There is lack of consensus around the management of these patients with conflicting evidence from non-randomised, retrospective studies of intensive chemotherapy regimens and a lack of randomised studies [19–21, 23]. Our data suggests that the majority of anthracycline eligible patients with *MYC*-*BCL2* DH/TH DLBCL receive standard R-CHOP, and a third receive DA-R-EPOCH or R-CODOX-M/R-IVAC. FISH results impacted on management as approximately half of all patients treated with ‘intensified’ regimens were switched from R-CHOP once FISH results defining a DH/TH translocation became available. Patients switched from RCHOP to intensive therapy were present across the majority of centres in our study (10/16) but were younger (median age of 60 vs 64 years, *P* = 0.05). Given the lack of consensus regarding the optimal treatment of these patients, patient age may have contributed to the decision to intensify therapy when FISH results became available.

In contrast to previous studies, we analysed *MYC*-*BCL2* DH/TH DLBCL cases separate from *MYC-BCL6* rearranged DLBCL. In keeping with previous observations, *MYC-BCL6* rearranged DLBCL constituted a minority of cases (10% of MYCR DLBCL cases) and were associated with superior survival compared to *MYC-BCL2* DH/TH cases. Intensified therapy (DA-R-EPOCH or R-CODOX-M/R-IVAC) in the *MYC-BCL2* DH/TH group was associated with improvements in TTNT and OS, when compared to R-CHOP like regimens. Interestingly, intensification of treatment appeared to have no effect, with a trend towards adverse outcomes within *MYC*-*BCL6* DLBCL cases in this study. The nature of this retrospective non-randomised study means biases relating to case acquisition and unmeasured confounders influencing treatment decisions and outcomes cannot be accounted for and thus tempers this study’s findings.

Nevertheless, these results corroborate the continued assessment for *MYC* translocations in DLBCL patients and suggest a role for treatment intensification in *MYC-BCL2* DH/TH cases. Testing for IG-MYC translocations may further refine prognostication [24]. There are however challenges with using FISH results for treatment selection including lack of a reliable histopathological marker to identify *MYC-BCL2* DH/TH cases thereby requiring routine FISH testing of all DLBCL cases and associated costs, increasing use of core biopsies resulting in scanty tissue (9% of cases in this study) and FISH turnaround times. Moreover, the morbidity and mortality associated with intensive chemotherapy in patients who are older or have a performance status ≥2, as demonstrated by the phase 2 study of R-CODOX-M/R-IVAC in patients with stage II-IV IPI ≥ 3 DLBCL, highlights the need for careful patient selection for an intensified chemotherapeutic approach [25].

Ultimately, less toxic therapies are needed for this high risk group of patients. The recently published randomised phase III POLARIX trial evaluating Polatuzumab Vedotin with R-CHP (Pola-R-CHP) demonstrated an improvement in progression-free survival with no increase in toxicity over R-CHOP in patients with intermediate to high-risk IPI DLBCL. However, no significant OS benefit was detected at the 24-month time-point and no observable benefit in DH/TH DLBCL [26]. With reference to recent advances in our understanding of DLBCL biology, the genomic clusters *MYC-BCL2* DH/TH cases best match to are EZB, C3 and BCL2, thus raising the prospect of less toxic, targeted therapies being developed to replace currently used intensified chemotherapy regimens. In contrast, *MYC-BCL6* DLBCL cases show a more variable gene expression and mutation profile [1, 5–7, 9].

In conclusion, our data supports the recent WHO and ICC recommendation of routine FISH testing of all aggressive B-cell lymphoma and separation of *MYC*-*BCL6* cases from *MYC*-*BCL2* DH/TH DLBCL cases to identify the highest risk cohorts. Within the limits of a retrospective analysis, our results suggest a possible role for treatment intensification in suitable *MYC-BCL2* DLBCL DH/TH cases until newer, more widely applicable, less toxic therapies can be identified.

## Supplementary information

Checklist

Supplementary Figures and Tables

## Data Availability

The data are not publicly available due to ethical restrictions.
