# Supplementary material for: A retrospective study of MYC rearranged diffuse large B-cell lymphoma in the context of the new WHO and ICC classifications
Source: Blood Cancer J. 2023 Apr 18;13(1):54. doi: 10.1038/s41408-023-00827-5 (PMC10113386; doi:10.1038/s41408-023-00827-5)
Supplement: Supplementary file 2 — Supplementary Figures and Tables [file 41408_2023_827_MOESM2_ESM.docx]

|  | | ***MYC-BCL2* DH/TH (n=115)** | **MYCR (n=60)** | ***MYC-BCL6* (n=21)** | ***P*-value*** | **R-CHOP**  **(n=95)** | **Intensive (n=44)** | ***P*-value** | ***MYC-BCL2* DH/TH + Intensive (n=26)** | ***MYC-BCL2* DH/TH + R-CHOP (n=65)** | ***P*-value** |
| --- | --- | --- | --- | --- | --- | --- | --- | --- | --- | --- | --- |
| **Age** | **Median (years)** | 65 | 68 | 68 | 0.43 | **65** | **56** | **0.002** | **55** | **66** | **0.009** |
|  | **Range (years)** | 28-87 | 37-84 | 27-89 |  | 33-84 | 28-79 |  | **28-79** | **39-84** |  |
| **Age groups (years)** | **10-29** | 1 | 1 | 0 |  | 0 | 1 |  | 1 | 0 |  |
|  | **30-49** | 11 | 5 | 3 |  | 9 | 8 |  | 5 | 6 |  |
|  | **50-69** | 63 | 27 | 11 |  | 55 | 30 |  | 16 | 38 |  |
|  | **70-89** | 40 | 22 | 7 |  | 31 | 5 |  | 4 | 21 |  |
|  | **>90** | 0 | 5 | 0 |  | 0 | 0 |  | 0 | 0 |  |
| **Sex** | **Male** | 69 | 38 | 11 | 0.99 | 54 | 32 | 0.11 | 18 | 36 | 0.33 |
|  | **Female** | 46 | 22 | 10 |  | 41 | 12 |  | 8 | 29 |  |
| **Performance status** | **0-1** | 60 | 28 | 8 | 0.48 | 49 | 29 | 0.44 | **19** | **31** | **0.05** |
|  | **≥2** | 37 | 21 | 7 |  | 28 | 12 |  | **4** | **21** |  |
|  | **Missing** | 18 | 11 | 6 |  | 18 | 3 |  | 3 | 13 |  |
| **Stage** | **1-2** | **13** | **16** | **2** | **0.01** | **21** | **3** | **0.01** | 1 | 10 | 0.10 |
|  | **3-4** | **92** | **34** | **14** |  | **64** | **41** |  | 25 | 49 |  |
|  | **Missing** | 10 | 10 | 5 |  | 10 | 0 |  | 0 | 6 |  |
| **Lactate dehydrogenase** | **<ULN** | 15 | 11 | 2 | 0.43 | 18 | 4 | 0.06 | 3 | 10 | 0.46 |
|  | **>ULN** | 74 | 32 | 14 |  | 54 | 35 |  | 20 | 39 |  |
|  | **Missing** | 26 | 17 | 5 |  | 23 | 5 |  | 3 | 16 |  |
| **CNS involvement at diagnosis** | **Present** | 3 | 2 | 1 |  | 0 | 3 |  | 2 | 0 |  |
| **International Prognostic Index** | **0-2** | 26 | 14 | 2 | 0.82 | 25 | 10 | 0.20 | 9 | 12 | 0.39 |
|  | **≥3** | 58 | 27 | 12 |  | 38 | 27 |  | 13 | 28 |  |
|  | **Missing** | 31 | 19 | 7 |  | 32 | 7 |  | 4 | 25 |  |

**Supplementary table 1: Baseline characteristics of subtypes.** DH, double-hit; TH, triple-hit; *MYCR*, MYC rearranged, ULN, upper limit of normal. *Represents testing between DH/TH and non-DH/TH cases. Age was considered in 5 categories (10-30 years, 30-50 years, 50-70 years, 70-90 years, 90-110 years); Eastern Cooperative Oncology Group (ECOG) performance status (PS) in 2 categories (0-1, 2-5); stage in 2 categories (1-2, 3-4); lactate dehydrogenase (LDH) in 2 categories (normal, >upper limit of normal (ULN)); and IPI in two categories (low, 0 to 2; high, 3 to 5).

| **Characteristic** | **Time to next treatment**  **(hazard ratio and 95% confidence interval)** | ***P*-value** | **Overall survival**  **(hazard ratio and 95% confidence interval)** | ***P*-value** |
| --- | --- | --- | --- | --- |
| **Age**  **(for an increase of 20 years)** | 1.19 (0.92-1.52) | 0.2 | **1.45 (1.09-1.95)** | **0.01** |
| **Sex (reference F)** | 1.08 (0.76-1.53) | 0.7 | 0.99 (0.67-1.46) | 0.9 |
| **ECOG PS (reference 0-1)** | **2.14 (1.46-3.14)** | **9×10^-5^** | **2.66 (1.71-4/10)** | **1×10^-5^** |
| **LDH ratio (reference normal)** | **2.87 (1.60-5.15)** | **4×10^-4^** | **2.62 (1.25-5.49)** | **0.01** |
| **Stage (reference 1-2)** | 1.51 (0.92-2.48) | 0.1 | **1.71 (0.96-3.04)** | **0.06** |
| **IPI (reference 0-2)** | **1.97 (1.21-3.20)** | **0.006** | **2.64 (1.46-4.80)** | **0.001** |

**Supplementary table 2: Univariate cox-regression analysis of baseline patient characteristics and overall survival or time to next treatment in patients with diffuse large B-cell lymphoma with double-hit, triple-hit and *MYC* rearranged diffuse large B-cell lymphoma.**

| **Characteristic** | **Time to next treatment**  **(hazard ratio and 95% confidence interval)** | ***P*-value** | **Overall survival**  **(hazard ratio and 95% confidence interval)** | ***P*-value** |
| --- | --- | --- | --- | --- |
| **Age**  **(for an increase of 20 years)** | 0.77 (0.52-1.15) | 0.2 | 0.76 (0.47-1.23) | 0.3 |
| **Sex (reference F)** | 0.99 (0.59-1.66) | 0.9 | 0.93 (0.51-1.68) | 0.8 |
| **ECOG PS (reference 0-1)** | 1.52 (0.85-2.72) | 0.2 | 1.65 (0.85-3.20) | 0.1 |
| **LDH ratio (reference normal)** | 2.03 (0.86-4.79) | 0.1 | 2.39 (0.84-6.76) | 0.1 |
| **Stage (reference 1-2)** | 1.31 (0.59-2.90) | 0.5 | 1.41 (0.55-3.58) | 0.5 |
| **IPI (reference 0-2)** | 1.53 (0.74-3.18) | 0.3 | 1.82 (0.77-4.32) | 0.2 |
| **R-CHOP vs intensive therapy (reference intensive therapy)** | **2.35 (1.25-4.44)** | **0.008** | **3.01 (1.34-6.77)** | **0.008** |

**Supplementary table 3: Univariate cox-regression analysis of baseline patient characteristics and overall survival or time to next treatment in patients with double-hit, triple-hit and *MYC* rearranged diffuse large B-cell lymphoma.**

| **Characteristic** | **Time to next treatment**  **(hazard ratio and 95% confidence interval)** | ***P*-value** | **Overall survival**  **(hazard ratio and 95% confidence interval)** | ***P*-value** |
| --- | --- | --- | --- | --- |
| **Age**  **(for an increase of 20 years) + R-CHOP vs intensive therapy (reference intensive therapy)** | **2.70 (1.41-5.19)** | **0.005** | **3.23 (1.43-7.34)** | **0.004** |
| **Age**  **(for an increase of 20 years) + ECOG PS + R-CHOP vs intensive therapy (reference intensive therapy)** | **3.04 (1.43-6.44)** | **0.01** | **3.97 (1.51-10.40)** | **0.005** |
| **IPI (reference 0-2) + R-CHOP vs intensive therapy (reference intensive therapy)** | **2.81 (1.27-6.20)** | **0.01** | **3.41 (1.26-9.20)** | **0.02** |

**Supplementary table 4: Multivariate cox-regression analysis of baseline patient characteristics and overall survival or time to next treatment in patients with *MYC*-*BCL2* double-hit and triple-hit diffuse large B-cell lymphoma. Hazard ratios and *P*-values refer to the effect of R-CHOP vs intensive therapy (reference intensive therapy).**

**Supplementary Figure 1: Kaplan-Meier estimates for (A) overall survival in all patients with *MYC*-*BCL6* diffuse large B-cell lymphoma stratified by chemotherapy regimens; (B) time to next treatment in all patients with *MYC*-*BCL6* diffuse large B-cell lymphoma stratified by chemotherapy regimens.**

**Supplementary Figure 2: Kaplan-Meier estimates for (A) overall survival in all patients with *MYC* rearranged diffuse large B-cell lymphoma stratified by chemotherapy regimens; (B) time to next treatment in all patients with *MYC* rearranged diffuse large B-cell lymphoma stratified by chemotherapy regimens.**

**Supplementary Figure 3: Kaplan-Meier estimates for (A) overall survival in patients with receiving platinum-based chemotherapy for relapsed refractory diffuse large B-cell lymphoma stratified by translocation status; (B) time to next treatment in patients with receiving platinum-based chemotherapy for relapsed refractory diffuse large B-cell lymphoma stratified by translocation status.**
